# Mental health issues in adolescent mothers and young adult mothers: the Brazilian context

**DOI:** 10.1016/j.jped.2025.03.011

**Published:** 2025-05-27

**Authors:** Amanda Ferreira de Carvalho, Daniela Dal Forno Kinalski Guaranha, Bruna Marmett, Júlia Mathias Reis, Carmem Lisiane Escouto de Souza, Bruna Silveira da Rosa, Tiago Chagas Dalcin, Sérgio Luis Amantea

**Affiliations:** a Ministério da Saúde, Associação Hospitalar Moinhos de Vento, Programa de Desenvolvimento Institucional do SUS (PROADI-SUS), Escritório de Projetos, Brazil; b Associação Hospitalar Moinhos de Vento, Instituto de Pesquisa, Porto Alegre, RS, Brazil

**Keywords:** Adolescent pregnancy, Pregnancy, Mental health, Depression, Anxiety

## Abstract

**Objective:**

To analyze anxiety and depression levels in adolescent and young adult mothers.

**Methods:**

This multicenter cross-sectional study was conducted across Brazil’s five regions. A non-probabilistic sample of 1177 adolescent (10–19 years) and young adult (20–29 years) mothers was selected proportionally based on regional live birth rates. Data collection (August 2022–May 2023) involved a structured questionnaire assessing mental health (DASS-21), family support, and quality of life. Statistical analyses were performed using RStudio©, with p-values < 0.05 considered significant. The study was approved by the Research Ethics Committee (CAAE: 55465822.0.2003.5086).

**Results:**

Depression or anxiety was reported by 22.7 % of both adolescent and young adult mothers, with 7.55 % and 7.07 % diagnosed during pregnancy, respectively. Among affected adolescents, 66.67 % did not seek follow-up care, primarily due to limited healthcare access (29.55 %). Adolescent mothers showed a higher prevalence of depression (*p* < 0.001), anxiety (*p* < 0.001), and stress (*p* = 0.027) postpartum. Depression was associated with a lack of emotional support from the child’s father (*p* = 0.096), insufficient family caregiving support (*p* = 0.014), and low perceived quality of life.

**Conclusion:**

Adolescent mothers exhibit greater vulnerability to mental disorders, emphasizing the need for targeted psychological and social support during the perinatal period.

## Introduction

Approximately 1 million births occur annually among adolescents under 15 years of age, while an additional 16 million births take place among those aged 15 to 19 years [1]. In low- and middle-income countries, 50 % of adolescent pregnancies are unplanned [2]. In Brazil, for every 1000 births, 68.4 are from girls aged 15 to 19, totaling 364,734 live births [3]. This number may be underestimated, as it represents only live births and does not account for stillbirths and abortions. Beyond concerns related to early motherhood indicators, there is increasing apprehension about the broader impact that teenage pregnancy can have on the lives of the affected girls [2].

An estimated one billion people worldwide live with a mental disorder (MD), with adolescents accounting for 14 % of this population [4]. Mental health conditions such as anxiety and depression are among the leading complications during and after pregnancy, affecting up to 20 % of mothers [5]. In Brazil, psychiatric hospitalizations among adolescents are more common in females and are often associated with mood disorders, such as depression and anxiety [6].

The causes of mental disorders like anxiety and depression are multifactorial. Nonetheless, risk factors for the development of MDs related to motherhood include being an adolescent, having higher parity, and having a history of prior or family depression, with up to 29 % of adolescent mothers diagnosed with depression [7]. Lack of social support, the adolescent's perception of her pregnancy, parental stress, and social isolation are variables that may contribute to the development of MDs as the adolescent mother progresses [5].

Given this context, this study aims to analyze anxiety and depression levels in adolescent and young adult mothers, seeking to understand differences and potential similarities between these populations to contribute to the development of support strategies and interventions for adolescent mothers. Secondary outcomes include assessing stress levels at the time of the interview and the association of mental health outcomes with variables such as family support, gestational health, and quality of life.

## Method

This multicenter, cross-sectional, descriptive, and quantitative study was conducted across Brazil’s five regions as part of the study “Vulnerabilities of Early Pregnancy in Brazil: Impacts on Adolescent Mothers and Children,” supported by PROADI-SUS. The study adhered to the STROBE checklist (Table S1 of Supplementary Material).

One reference hospital per region was selected based on high adolescent birth rates (SINASC) [3]. Hospitals linked to the Unified Health System (SUS) and certified by the Baby-Friendly Hospital Initiative (BFHI) provided lists of mothers who gave birth from June 2021 to June 2022. Data collection occurred from August 2022 to May 2023, including 1177 adolescent (10–19 years) and young adult (20–29 years) mothers, selected alphabetically from hospital records and invited via telephone.

Participants were selected in an orderly fashion, following the alphabetical list of mothers who gave birth in the study's partner hospitals. All participants who met the inclusion criteria were invited to participate in the study via telephone contact.

The inclusion criteria were: mothers aged 10 to under 20 years old and mothers aged 20 to under 30 years old who gave birth at the selected institutions; signed the Free and Informed Consent Form (ICF) or the Free and Informed Assent Form (IAF); were fluent in Brazilian Portuguese; were able to understand the study objectives; and had stayed with their baby after birth. Additionally, mothers who gave birth at least six months before the interview were prioritized to account for the timeframe of postpartum disorders, such as postpartum depression, ensuring that potential mental disorder symptoms were not related to the puerperium, defined as the period from birth to eight weeks postpartum [8].

Data collection was conducted through in-person interviews at the participants’ residences or during routine consultations at hospital services.

Demographic, mental health (including diagnoses, follow-up, and treatments), gestational health, family support, and quality of life variables were assessed, all included in the questionnaire developed by the authors. These variables are detailed in Table S3 of the supplementary material. Mental health variables identifying symptoms of depression, anxiety, and stress were evaluated using the Depression, Anxiety, and Stress Scale (DASS-21), which includes statements based on participants’ experiences. Interviews were conducted at least six months postpartum, and the DASS-21 scale evaluates symptoms over the previous two weeks, contributing to the accuracy of anxiety, depression, and stress levels related to the post-puerperium period. Other domains and questionnaire information can be found in the supplementary material (Table S2-S5).

The Kaiser-Meyer-Olkin (KMO) test result was 0.949, indicating high model quality. Cronbach's alpha values were 0.92 for depression, 0.90 for stress, and 0.86 for anxiety, reflecting satisfactory internal consistency for each component. Factor analysis and the distribution of factors among subscales indicated that the structure with three distinct factors is appropriate [9].

To analyze adequate education levels among adolescent and young adult mothers, the Brazilian legislation defining appropriate age ranges for schooling was used as a reference, where high school should start at age 15 and end at age 17 [10].

DASS-21 levels are categorized as normal, mild, moderate, severe, and extremely severe [11]. For this study, the “normal” level was defined as participants without any symptoms of depression, anxiety, or stress, while other levels were classified as having symptoms. Thus, DASS-21 results are presented both as the presence or absence of symptoms (yes/no) and as different levels (normal, mild, moderate, severe, and extremely severe). Quality of life was assessed using the shortened version of the World Health Organization Quality of Life Questionnaire (WHOQOL-BREF) [12].

The sample size was calculated using RStudio© software (version 2022.12.0 + 353, 2022 by Posit Software, PBC). A total of 1146 participants was estimated, based on a 26.3 % prevalence of anxiety and depression, a 10 % potential loss, 80 % statistical power, and a 5 % significance level [13]. Sampling was non-probabilistic, with proportional inclusion of adolescent mothers according to the regional distribution of live births in Brazil.

Data analysis involved database creation, coding, and storage in Excel spreadsheets. To ensure data quality and reliability, a double-entry process was conducted, followed by validation.

Descriptive analyses of categorical variables (sociodemographic and mental health) were performed using absolute (n) and relative (%) frequencies. The continuous variable of age was described as the median and interquartile range (Q1–Q3). The Shapiro-Wilks test was used to analyze the normality of variable distribution. Comparisons between categorical variables for adolescent and young adult mothers were performed using the Chi-Square test, while the Mann-Whitney test was used for numerical variables.

For the mental health questionnaire (DASS-21), which is a Likert-type scale ranging from 0 (Did not apply to me at all) to 3 (Applied to me very much or most of the time), scores were calculated for each domain (Depression, Anxiety, and Stress). Scores were obtained by summing item responses within each domain and multiplying the total by 2. Based on the final score, each domain was classified as normal, mild, moderate, severe, or extremely severe [11]. Cronbach's alpha coefficient was used to evaluate the reliability of the DASS-21 questionnaire.

Associations were analyzed using Poisson regression models with robust variance correction, a preferred approach for estimating Prevalence Ratios compared to logistic regression [14]. To examine the associations of each DASS-21 domain with gestational support, gestational health, and quality of life variables, new variables were created for each domain (Depression, Anxiety, and Stress) indicating whether the participant had any level above mild. Models were constructed using these dichotomous variables as dependent variables, while gestational support, gestational health, and quality of life variables were used as independent variables. Group (Adolescent Mother or Young Adult Mother) was used as a potential confounder. Results are presented as Prevalence Ratios (PR) with 95 % Confidence Intervals (CI). P-values < 0.05 were considered statistically significant.

Data contributing to this study were analyzed using RStudio© software (version 2022.12.0 + 353, 2022 by Posit Software, PBC).

The questionnaire included options for “do not want to answer” or “do not remember the answer,” and participants selecting these were excluded from the respective variable analysis. In multiple-choice questions, responses could exceed the number of participants, leading to variations in response counts across variables.

The study was approved by the Research Ethics Committee (CAAE: 55465822.0.1001.5330), following Resolution 466/12/CNS/MS [15]. Legal guardians provided consent for underage participants, who also gave assent.

## Results

This study included 1177 mothers, 583 adolescents and 594 young adults. Sociodemographic and mental health variables are presented in Table 1. Adolescent mothers and young adult mothers had a median age of 18 years (16–19) and 24 years (22–26), respectively. 62.95 % of adolescents and 57.24 % of young adults identified themselves as mixed race ("pardo"). Concerning education, 35.85 % of adolescents did not complete high school, and 49.22 % were under 17 years old, suggesting inadequate education for 23.16 %. Over 16 years old, 91.5 % of adolescent mothers were unemployed.

**Table 1:** Description and comparison of mental health variables of adolescent and young adult mothers.

Variables	Adolescent mothers *n* = 583	Young adult mothers *n* = 594	*p*-value^a^
*Mental health variables*			
Diagnosis of depression or anxiety			0.9267
No	451 (77.36)	459 (77.27)	
Yes, prior to the pregnancy of 2021/2022	88 (15,09)	93 (15,66)	
Yes, During the pregnancy of 2021/2022	44 (7,55)	42 (7,07)	
Professional counseling for depression and/or anxiety			0.6827
Yes	21 (15,91)	18 (13,33)	
No	88 (66,67)	93 (68,89)	
Sporadically, only when experiencing an issue	15 (11,36)	19 (14,07)	
Never was	8 (6,06)	5 (3,7)	
Main reasons for not seeking professional counseling			0.4595
Lack of time	2 (2,27)	4 (4,30)	
Failed to access a psychiatrist through the public healthcare system (SUS)	2 (2,27)	6 (6,45)	
Failed to access a psychologist through the public healthcare system (SUS)	26 (29,55)	26 (27,96)	
Not encouraged to pursue counseling	23 (26,14)	27 (29,03)	
Lack of awareness about the importance of counseling	5 (5,68)	8 (8,6)	
Lack of interest	24 (27,27)	20 (21,51)	
No health insurance	4 (4,55)	1 (1,08)	
Don't know	1 (1,14)	–	
No one to leave the child with	1 (1,14)	1 (1,08)	
Use of medication for anxiety			0.3916
Yes, and purchased	11 (25)	8 (19,05)	
Yes, and obtained through SUS	2 (4,55)	7 (16,67)	
Yes, but not used	4 (9,09)	1 (2,38)	
No	27 (61.36)	26 (61.9)	
Use of medication for depression			0.0846
Yes, and purchased	2 (4,55)	5 (11,9)	
Yes, and obtained through SUS	1 (2,27)	3 (7,14)	
Yes, but not used	–	3 (7,14)	
No	41 (93.18)	31 (73.81)	

The results were presented as n (%). Categorical variables were evaluated using the chi-square test.

a Statistically significant estimate *p* < 0.05.

In terms of mental health, 132 adolescent mothers (22.7 %) and 135 young adult mothers (22.7 %) had been diagnosed with depression or anxiety at some point in their lives. During pregnancy, these conditions were diagnosed in 7.55 % of adolescents and 7.07 % of young adults. Notably, most affected individuals — 66.67 % of adolescents and 68.89 % of young adults — did not receive follow-up care for depression and/or anxiety. The primary barriers were difficulty accessing psychologists through the SUS (reported by 29.55 % of adolescents and 27.96 % of young adults) and lack of motivation to continue care (25.14 % and 29.03 %, respectively). Among those diagnosed during pregnancy, the majority did not require medication for anxiety (61.36 % of adolescents and 61.9 % of young adults) or depression (93.18 % of adolescents and 73.81 % of young adults) (Table 1).

**Figure 1:**
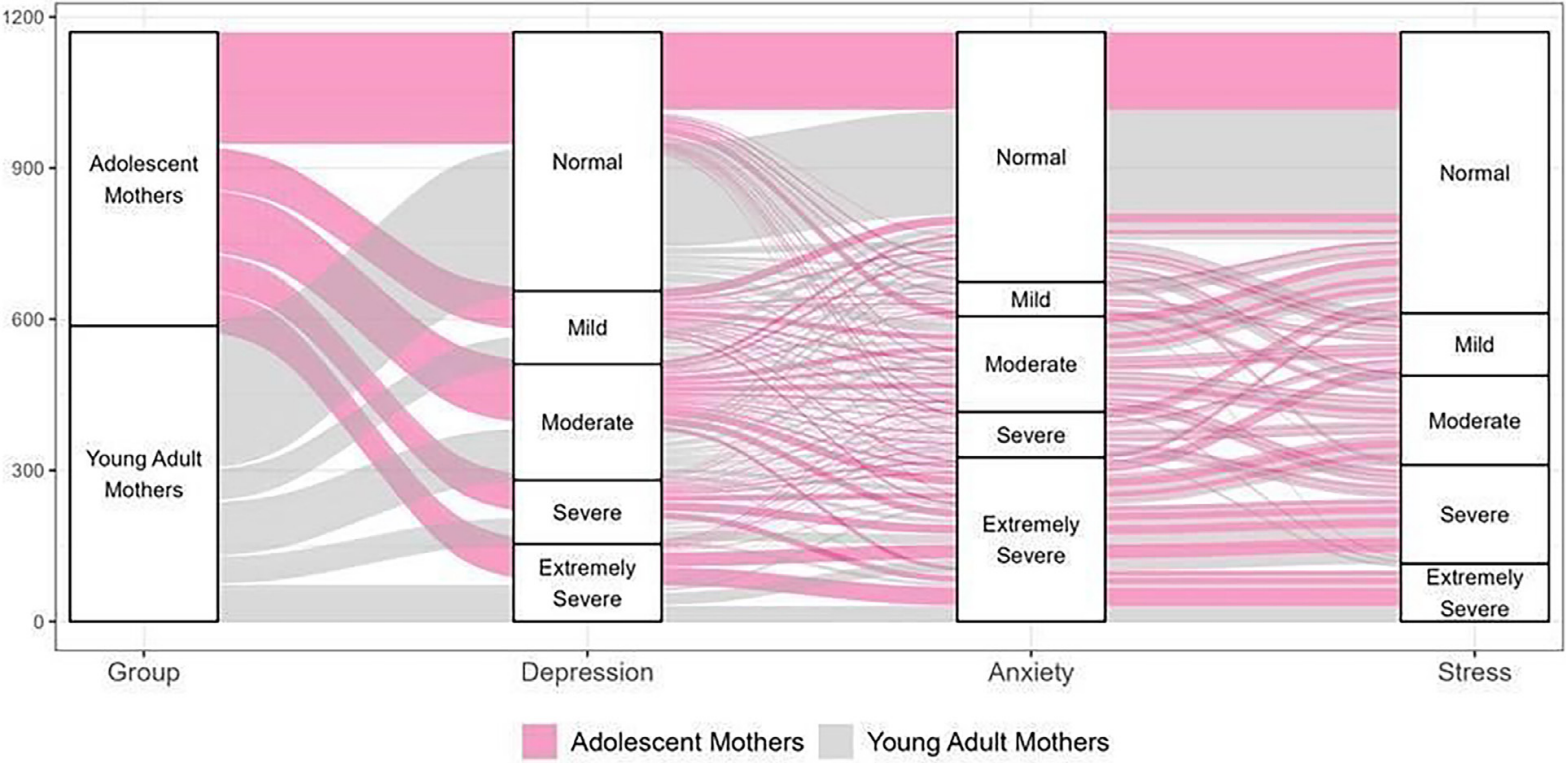
Relationship of the levels obtained in the DASS-21 for depression, anxiety, and stress observed in the groups of adolescent and young adult mothers. Source: Authors' elaboration, 2023.

The DASS-21 scale demonstrated high reliability, with a Cronbach's alpha of 0.94 overall and subscale scores of 0.83 for depression, 0.86 for anxiety, and 0.85 for stress, indicating strong internal consistency. Figure 1 presents the distribution of scores, highlighting group differences. Briefly, the most frequent outcomes included 154 adolescent mothers (26.42 %) and 207 young adult mothers (34.85 %) scoring within the normal range for depression, anxiety, and stress, while 36 adolescent mothers (6.17 %) and 32 young adult mothers (5.39 %) displayed extremely severe levels of these conditions.

Adolescent mothers exhibited higher levels of depression, anxiety, and stress compared to young adult mothers at the time of the interview (Table 2). Among those without emotional support from the child’s father or family, the prevalence of depression was 20 % higher. Conversely, mothers receiving emotional support or caregiving assistance experienced a 16 % lower prevalence of depression and a 15 % lower prevalence of anxiety. Additional factors, such as leaving their parents’ homes or having partners resistant to contraceptive use, were linked to an 18 % higher prevalence of anxiety and a 32 % higher prevalence of stress. Furthermore, significant life changes post-pregnancy, such as losing friendships or experiencing family rejection, increased the prevalence of depression by 34 %, anxiety by 18 %, and stress by 32 %.

**Table 2:** Levels of depression, anxiety, and stress in adolescent and young adult mothers through the DASS-21.

	**Adolescent mothers *n*=583**	**Young adult mothers *n*=594**	***p*-value**
DASS-21 condition			
Depression			<0001^a^
Yes	361 (61,92)	300 (50,5)	
No	222 (38,07)	294 (49,49)	
Anxiety			<0001^a^
Yes	372 (63,8)	307 (51,68)	
No	211 (36,2)	287 (48,3)	
Stress			0027^a^
Yes	325 (55,74)	292 (49,15)	
No	258 (44,25)	302 (50,84)	
Depression/Anxiety/Stress			
Yes	266 (45,63)	211 (35,52)	<0001^a^
No	317 (54,37)	383 (64,48)	

DASS-21 Levels			

Depressão			0002^a^
Normal	222 (38,08)	294 (49,49)	
Mild	83 (14,24)	62 (10,44)	
Moderate	124 (21,27)	108 (18,18)	
Severe	73 (12,52)	55 (9,26)	
Extremely severe	81 (13,89)	75 (12,63)	
Anxiety			<0001^a^
Normal	211 (36,19)	287 (48,32)	
Mild	32 (5,49)	37 (6,23)	
Moderate	103 (17,67)	88 (14,81)	
Severe	48 (8,23)	42 (7,07)	
Extremely severe	189 (32,42)	140 (23,53)	
Stress			0128
Normal	258 (44,25)	302 (50,84)	
Mild	70 (12,01)	54 (9.09)	
Moderate	87 (14,92)	91 (15,32)	
Severe	104 (17,84)	95 (15,99)	
Extremely severe	64 (10,98)	52 (8,75)	

The data are presented as median (Q1-Q3) and absolute frequency (%). Comparison between groups was performed using the chi-square test.

a A *p*-value <0.05 was considered statistically significant.

When analyzing physical and psychological quality of life, a slight improvement in physical quality of life corresponded to a marginal 1 % decrease in anxiety and stress levels. However, no significant relationship was observed between physical quality of life and depression. In contrast, enhanced psychological quality of life was associated with a 2 % reduction in depression, anxiety, and stress levels. These findings underscore the critical role of emotional support and quality of life in shaping the mental health outcomes of adolescent and young adult mothers. The associations between quality of life and mental health outcomes are detailed in Table 3.

**Table 3:** Association of depression, anxiety, and stress with variables of family support, gestational health, and quality of life of adolescent and young adult mothers.

	Depression		Anxiety		Stress	
	RP (IC 95 %)	Valor de p	RP (IC 95 %)	Valor de p	RP (IC 95 %)	Valor de p
Family support						

Emotional support from the child's father						
No	1.00	–	1.00	–	1.00	–
Yes	0,8 (0,67–0,95)	0,0096	0,87 (0,73–1,03)	0,0959	0,88 (0,72–1,07)	0,1914
Activities of daily living (routine)						
No	1.00	–	1.00	–	1.00	–
Yes	0,84 (0,73–0,96)	0014	0,92 (0,79–1,07)	0285	0,95 (0,79–1,13)	0558
Healthcare practices						
No	1.00	–	1.00	–	1.00	–
Yes	0,95 (0,82–1,10)	0,4921	0,85 (0,74–0,98)	0,0212	0,81 (0,69–0,95)	0,0098

Gestational health–reasons for becoming pregnant						

Moving out of parents' house^a^						
No	1.00	–	1.00	–	1.00	–
Yes	1,14 (0,97–1,34)	0,1076	1,18 (1,03–1,36)	0,0147	–	–
Partner didn't want to use a condom						
No	1.00	–	1.00	–	1.00	–
Yes	1,10 (0,99–1,23)	0,0731	1,08 (0,97–1,20)	0,1659	1,13 (1,00–1,28)	0,0415

Gestational health–Life changes resulting from pregnancy						

Life became more challenging						
No	1.00	–	1.00	–	1.00	–
Yes	1,34 (1,17–1,52)	<0001	1,18 (1,05–1,33)	0005	1,32 (1,15–1,52)	<0001^b^
Was rejected by family						
No	1.00	–	1.00	–	1.00	–
Yes	1,06 (0,92–1,22)	0386	1,16 (1,02–1,32)	0,0243	1,07 (0,91–1,26)	0,3875
Improved self-esteem						
No	1.00	–	1.00	–	1.00	–
Yes	0,84 (0,75–0,93)	<0001	0,88 (0,79–0,98)	0,0242	0,84 (0,75–0,94)	0,0032
Lost friends/school peer group						
No	1.00	–	1.00	–	1.00	–
Yes	1,26 (1,13–1,40)	<0001	1,21 (1,09–1,35)	<0001	1,30 (1,16–1,47)	<0001^b^
Dating became more challenging						
No	1.00	–	1.00	–	1.00	–
Yes	1,12 (1,01–1,25)	0026	1,06 (0,96–1,17)	0,2814	1,17 (1,04–1,31)	0,0076

Quality of life						

Physical	1,0 (0,99–1,00)	0014	0,99 (0,99–1,00)	0,0021	0,99 (0,99–1,00)	<0001^b^
Psychological	0,98 (0,98–0,99)	<0001	0,99 (0,99–1,00)	<0001	0,98 (0,98–0,99)	<0001^b^
Social relationships	1,0 (0,99–1,00)	0,0234	1,0 (1,0–1,0)	0,5883	1,0 (1,0–1,0)	0,3061
Environment	1,0 (0,99–1,00)	0,5735	1,0 (0,99–1,0)	0,2159	1,0 (0,99–1,00)	0,9158

Data are presented as prevalence ratio (95 % CI). Data analysis was performed with comparison between groups and was performed using the chi-square test.

a Variable showed no association and was not included in the model.

b *p* < 0,05 was considered statistically significant.

## Discussion

The findings of this study demonstrated an alarming reality regarding the mental health of adolescent mothers in Brazil, highlighting the relationship between socioeconomic conditions and the prevalence of emotional symptoms. A higher association of depressive, anxious, and stress symptoms was observed in the adolescent group after the birth of their child, whereas young adult mothers exhibited a greater prevalence of normal levels of these symptoms. Adolescent mothers, however, showed a stronger association with severe cases of these conditions. The high prevalence of emotional distress was especially notable among adolescent mothers who lacked emotional support from the child’s father. Significant life changes after pregnancy, such as the loss of friendships or family rejection, also contributed to the increased prevalence of emotional symptoms.

The analysis of physical quality of life showed that a slight increase in physical quality of life was associated with a minimal reduction in the prevalence of anxious and stress symptoms, though no significant association was found between physical quality of life and depression levels. In contrast, an improvement in psychological quality of life was associated with a decrease in depression, anxiety, and stress symptoms.

Adolescent pregnancy impacts the mental health of mothers. Adolescent mothers represent a vulnerable group due to the transformations that characterize adolescence, including behavioral, social, and motherhood-related changes [16]. It was identified that 22.7 % of adolescents and 22.7 % of young adult mothers had been diagnosed with depression or anxiety before or during pregnancy. It is worth noting that the prevalence of psychiatric symptoms during pregnancy varies between 10 % and 16 % in the adolescent population, meaning at least one in four pregnant adolescents experiences such disorders [17,18]. Among young adult mothers, a study conducted in São Paulo estimated that 26.6 % met the criteria for mental disorders, with 14.4 % likely diagnosed with depression.¹⁹ However, this study evaluated mental health conditions during pregnancy, differing from the postpartum period assessed in this study.

Participants in this study were assessed for the presence of probable mental disorders after the puerperal period.⁸ This analysis is innovative in the Brazilian context, as previous studies typically focus on evaluation during pregnancy or immediately after childbirth [17,19, 20, 21]. Adolescent mothers showed a higher prevalence of depression, anxiety, and stress levels compared to young adult mothers. Another study found that 26.6 % of mothers exhibited changes consistent with probable mental disorders, with some level of depression in 16.2 % and some level of anxiety in 20.4 % [22]. In international studies, the prevalence of mental disorders, particularly anxiety and depression, ranges from 11.8 % to 34.9 % [22,23].

Among mothers diagnosed with depression and anxiety, more than half of the adolescents (66.67 %) and young adults (68.89 %) did not receive treatment for their condition. Specialized literature reveals that 48 % of mothers experiencing anxiety and 70 % of those experiencing depression remain in psychological distress due to the lack of specialized guidance and support [20]. Many mothers do not seek help for their symptoms due to stigma or limited access to resources, resulting in low adherence to treatment [24]. A Brazilian study conducted in 2017 revealed that social support provided to mothers during pregnancy, regardless of age group, can reduce depression levels after childbirth. Therefore, the support of healthcare teams and the child’s father are protective factors against mental health issues [25]. However, it should be noted that the aforementioned study evaluated participants within 48 hours postpartum, representing a significant difference from the present analysis.

It was identified that mothers with some level of depression faced a lack of emotional support from their partner and family in daily caregiving, while those with anxiety and stress also did not receive family support for their health. In Brazil, a lack of partner support during pregnancy increases the risk of mental disorders in adolescents by 1.4 times [26]. After pregnancy, these mothers face additional difficulties, such as depression, anxiety, and stress levels associated with loss of self-esteem and social networks. Family rejection and relationship issues also contribute to these disorders, emphasizing the importance of family support for the mental health of adolescent mothers [27,28]. Adolescents who become mothers and have little or no family support experience low levels of self-esteem and self-efficacy, making them more prone to depressive symptoms [5].

This study presented some limitations that should be considered, such as data collection in only five capital cities, which limited its national representativeness. The data were obtained exclusively from institutions certified by the “Baby-Friendly Hospital Initiative (BFHI),” which may not reflect the reality of all pregnancies in Brazil. Additionally, the sample size calculation was based on international studies, affecting territorial representativeness. Interviews were self-reported, which could introduce recall bias. Most mothers were pregnant during the COVID-19 pandemic, which could have impacted access to healthcare and mental health.

Early pregnancy significantly impacts adolescents' mental health, with higher rates of depression, anxiety, and stress. Family support, gestational health, and quality of life are key influencing factors. Adolescent mothers' vulnerability to mental disorders underscores the need for targeted health interventions. By assessing mental health beyond pregnancy and postpartum, this study offers a unique perspective in Brazil, providing a foundation for public policies to support adolescent mothers and their families.

## Authors' contributions

Carvalho AF, Marmett B, Guaranha DK, Reis JM, Rosa BS, Souza CLE, Dalcin TC, Amantéa SL contributed to the study design, project conception, data analysis, and interpretation; AF, Marmett B, Guaranha DK, Reis JM, Rosa BS, Souza CLE, Dalcin TC, Amantéa SL: Drafting of the manuscript and critical revision of its intellectual content. All authors approved the final version of the manuscript and are responsible for all its aspects, including the assurance of its accuracy and integrity.

## Funding

Financial support was provided by the Associação Hospitalar Moinhos de Vento in partnership with the Ministry of Health, through the Programa de Desenvolvimento Institucional do Sistema Único de Saúde (PROADI-SUS).

## Conflicts of interest

The authors declare no conflicts of interest.

## Appendix Supplementary materials

Image, application 1

## Footnotes

Supplementary material associated with this article can be found in the online version at doi:10.1016/j.jped.2025.03.011.
