# Supplementary material for: Mental health issues in adolescent mothers and young adult mothers: the Brazilian context
Source: J Pediatr (Rio J). 2025 May 27;101(4):608–15. doi: 10.1016/j.jped.2025.03.011 (PMC12276611; doi:10.1016/j.jped.2025.03.011)
Supplement: Supplementary file 1 [file mmc1.docx]

**JPED-D-24-00204_Supplementary Material**

**Mental health issues in Adolescent Mothers and Young Adult Mothers: The Brazilian context**

This cross-sectional study was conducted according to STROBE guidelines. The checklist is fulfilled as follows.

**Tabela S1**. STROBE Statement—checklist of items that should be included in reports of observational studies.

|  | **Item No.** | | **Recommendation** | **Page  No.** | | | **Relevant text from manuscript** |
| --- | --- | --- | --- | --- | --- | --- | --- |
| **Title and abstract** | | 1 | (*a*) Indicate the study’s design with a commonly used term in the title or the abstract | | 1 | |  |
|  |  |  | (*b*) Provide in the abstract an informative and balanced summary of what was done and what was found | | 2 | |  |
| **Introduction** | | | | | | |  |
| Background/rationale | | 2 | Explain the scientific background and rationale for the investigation being reported | | 3 -4 | |  |
| Objectives | | 3 | State specific objectives, including any prespecified hypotheses | | 4 | |  |
| **Methods** | | | | | | |  |
| Study design | | 4 | Present key elements of study design early in the paper | | 4 | |  |
| Setting | | 5 | Describe the setting, locations, and relevant dates, including periods of recruitment, exposure, follow-up, and data collection | | 5 | |  |
| Participants | | 6 | (*a*) *Cohort study*—Give the eligibility criteria, and the sources and methods of selection of participants. Describe methods of follow-up  *Case-control study*—Give the eligibility criteria, and the sources and methods of case ascertainment and control selection. Give the rationale for the choice of cases and controls  *Cross-sectional study*—Give the eligibility criteria, and the sources and methods of selection of participants | | 5-6 | |  |
|  |  |  | (*b*) *Cohort study*—For matched studies, give matching criteria and number of exposed and unexposed  *Case-control study*—For matched studies, give matching criteria and the number of controls per case |  | | |  |
| Variables | | 7 | Clearly define all outcomes, exposures, predictors, potential confounders, and effect modifiers. Give diagnostic criteria, if applicable | | | 5 |  |
| Data sources/ measurement | | 8* | For each variable of interest, give sources of data and details of methods of assessment (measurement). Describe comparability of assessment methods if there is more than one group | | | 5-6 |  |
| Bias | | 9 | Describe any efforts to address potential sources of bias | | | 5 |  |
| Study size | | 10 | Explain how the study size was arrived at | | | 4-5 |  |

| Quantitative variables | 11 | Explain how quantitative variables were handled in the analyses. If applicable, describe which groupings were chosen and why | 5-6 |  |
| --- | --- | --- | --- | --- |
| Statistical methods | 12 | (*a*) Describe all statistical methods, including those used to control for confounding | 5-6 |  |
|  |  | (*b*) Describe any methods used to examine subgroups and interactions | 5-6 |  |
|  |  | (*c*) Explain how missing data were addressed | 5-6 |  |
|  |  | (*d*) *Cohort study*—If applicable, explain how loss to follow-up was addressed  *Case-control study*—If applicable, explain how matching of cases and controls was addressed  *Cross-sectional study*—If applicable, describe analytical methods taking account of sampling strategy | 5-6 |  |
|  |  | (*It is*) Describe any sensitivity analyses | THAT |  |
| **Results** | | | | |
| Participants | 13* | (a) Report numbers of individuals at each stage of study—eg numbers potentially eligible, examined for eligibility, confirmed eligible, included in the study, completing follow-up, and analysed | 7 |  |
|  |  | (b) Give reasons for non-participation at each stage | 5-6 |  |
|  |  | (c) Consider use of a flow diagram | THAT |  |
| Descriptive data | 14* | (a) Give characteristics of study participants (eg demographic, clinical, social) and information on exposures and potential confounders | 7 |  |
|  |  | (b) Indicate number of participants with missing data for each variable of interest | Page 7-8, Table S2 - Supplementary material |  |
|  |  | (c) *Cohort study*—Summarise follow-up time (eg, average and total amount) |  |  |
| Outcome data | 15* | *Cohort study*—Report numbers of outcome events or summary measures over time |  |  |
|  |  | *Case-control study—*Report numbers in each exposure category, or summary measures of exposure |  |  |
|  |  | *Cross-sectional study—*Report numbers of outcome events or summary measures | 7, 10 - 14 |  |
| Main results | 16 | (*a*) Give unadjusted estimates and, if applicable, confounder-adjusted estimates and their precision (eg, 95% confidence interval). Make clear which confounders were adjusted for and why they were included | 14 |  |
|  |  | (*b*) Report category boundaries when continuous variables were categorized | THAT |  |
|  |  | (*c*) If relevant, consider translating estimates of relative risk into absolute risk for a meaningful time period | THAT |  |

| Other analyses | 17 | Report other analyses done—eg analyses of subgroups and interactions, and sensitivity analyses | 10 - 14 |  |
| --- | --- | --- | --- | --- |
| **Discussion** | | | | |
| Key results | 18 | Summarize key results with reference to study objectives | 14-18 |  |
| Limitations | 19 | Discuss limitations of the study, taking into account sources of potential bias or imprecision. Discuss both direction and magnitude of any potential bias | 19 |  |
| Interpretation | 20 | Give a cautious overall interpretation of results considering objectives, limitations, multiplicity of analyses, results from similar studies, and other relevant evidence | 20 - 24 |  |
| Generalisability | 21 | Discuss the generalisability (external validity) of the study results | 15-18 |  |
| **Other information** | |  | | |
| Funding | 22 | Give the source of funding and the role of the funders for the present study and, if applicable, for the original study on which the present article is based | 1 |  |

*Give information separately for cases and controls in case-control studies and, if applicable, for exposed and unexposed groups in cohort and cross-sectional studies.

**Note:** An Explanation and Elaboration article discusses each checklist item and gives methodological background and published examples of transparent reporting. The STROBE checklist is best used in conjunction with this article (freely available on the Web sites of PLoS Medicine at http://www.plosmedicine.org/, Annals of Internal Medicine at http://www.annals.org/, and Epidemiology at http://www.epidem.com/). Information on the STROBE Initiative is available at www.strobe-statement.org.

The analysis of biological, psychological and social domains by region are described in the tables below.

**Table S2**. Biological domains of adolescent mothers and young adult mothers in the five regions of Brazil.

| **Variable** | **South region** | | **Southeast region** | | **Midwest region** | | **Northeast Region** | | **North region** | |
| --- | --- | --- | --- | --- | --- | --- | --- | --- | --- | --- |
|  | Teen Moms^a^ | Young adult mothers^b^ | Teen Moms^a^ | Young adult mothers^b^ | Teen Moms^a^ | Young adult mothers^b^ | Teen Moms^a^ | Young adult mothers^b^ | Teen Moms^a^ | Young adult mothers^b^ |
| **Health History** | | | | | | | | | | |
| ***Health comorbidities*** |  |  |  |  |  |  |  |  |  |  |
| Diabetes | 3 (4.84) | 1 (1.67) | 3 (1.66) | 3 (1.69) | 1 (2.04) | 1 (2.13) | 2 (1.03) | 4 (1.9) | 1 (1.04) | 2 (2.02) |
| Hypertension | 1 (1.61) | 3 (5) | 8 (4.42) | 13 (7.3) | 1 (2.04) | 4 (8.51) | 2 (1.03) | 9 (4.29) | - | 3 (3.03) |
| Obesity | - | 2 (3.33) | - | 6 (3.37) | - | 2 (4.26) | 2 (1.03) | 13 (6.19) | - | 4 (4.04) |
| Malnutrition | - | - | 2 (1.1) | 2 (1.12) | 1 (2.04) | - | 4 (2.05) | 1 (0.48) | - | 1 (1.01) |
| Cardiovascular diseases | - | - | - | - | - | - | - | 2 (0.95) | - | - |
| Pneumopathies | 3 (4.84) | - | 1 (0.55) | 2 (1.12) | - | - | 3 (1.54) | 6 (2.86) | - | 2 (2.02) |
| Nephropathies | - | - | - | - | 1 (2.04) | - | 2 (1.03) | 1 (0.48) | 1 (1.04) | 2 (2.02) |
| Hematological | 1 (1.61) | - | 3 (1.66) | 5 (2.81) | - | - | 10 (5.13) | 5 (2.38) | 3 (3.12) | - |
| Neurological | 2 (3.23) | - | - | 1 (0.56) | - | - | 1 (0.51) | 5 (2.38) | - | 2 (2.02) |
| Autoimmune | - | - | - | - | - | - | 1 (0.51) | 1 (0.48) | - | 2 (2.02) |
| Infectious | 1 (1.61) | 2 (3.33) | 7 (3.87) | 8 (4.49) | - | - | 1 (0.51) | 2 (0.95) | 3 (3.12) | 1 (1.01) |
| Inflammatory | 2 (3.23) | 1 (1.67) | 2 (1.1) | 3 (1.69) | - | - | 2 (1.03) | - | - | - |
| Neoplasms | - | - | - | 1 (0.56) | - | - | - | - | - | - |
| Depression | 8 (12.9) | 10 (16.67) | 17 (9.39) | 23 (12.92) | 7 (14.29) | 2 (4.26) | 12 (6.15) | 17 (8.1) | 10 (10.42) | 6 (6.06) |
| Anxiety | 17 (27.42) | 16 (26.67) | 39 (21.55) | 32 (17.98) | 14 (28.57) | 8 (17.02) | 40 (20.51) | 35 (16.67) | 15 (15.62) | 18 (18.18) |
| Mental health disorder | 1 (1.61) | - | - | - | - | - | 1 (0.51) | 1 (0.48) | - | - |
| Adverse conditions | - | - | - | 1 (0.56) | - | - | 1 (0.51) | 2 (0.95) | - | 1 (1.01) |
| None | 40 (64.52) | 37 (61.67) | 125 (69.06) | 117 (65.73) | 33 (67.35) | 34 (72.34) | 131 (67.18) | 137 (65.24) | 72 (75) | 66 (66.67) |
| **Medication use^d^** | 17 (27.42) | 26 (43.33) | 61 (33.7)* | 81 (45.51) | 12 (24.49) | 16 (34.04) | 44 (22.56) | 66 (31.58) | 27 (28.12) | 22 (22.22) |
| **Main medicine (contraceptive)^c^** | 14 (22.58) | 23 (38.33) | 58 (32.04) | 70 (39.33) | 10 (20.41) | 15 (31.91) | 36 (18.46) | 51 (24.29) | 22 (22.92) | 18 (18.18) |
| **Visited a health service <2 weeks ago^d^** | 6 (9.68) | 13 (21.67) | 41 (22.91) | 26 (15.12) | 4 (8.33)* | 9 (19.15) | 30 (15.38) | 22 (10.58) | 17 (17.89) | 9 (9.09) |
| **Reason for consultations in health services** |  |  |  |  |  |  |  |  |  |  |
| Accident or injury | 3 (4.84) | - | 3 (1.66) | 2 (1.12) | 2 (4.08) | 3 (6.38) | 3 (1.54) | 6 (2.86) | 1 (1.04) | 3 (3.03) |
| Continuation of treatment or therapy | 3 (4.84) | 6 (10) | 4 (2.21) | 8 (4.49) | 3 (6.12) | 1 (2.13) | 8 (4.1) | 15 (7.14) | 3 (3.12) | 6 (6.06) |
| Prenatal consultation | 14 (22.58) | 10 (16.67) | 35 (19.34) | 38 (21.35) | 7 (14.29) | 6 (12.77) | 63 (32.31) | 43 (20.48) | 25 (26.04) | 20 (20.2) |
| Routine tests | 24 (38.71) | 25 (41.67) | 48 (26.52) | 66 (37.08) | 24 (48.98) | 16 (34.04) | 64 (32.82) | 91 (43.33) | 60 (62.5) | 54 (54.55) |
| Sexually transmitted infection | 1 (1.61) | 1 (1.67) | 5 (2.76) | - | 1 (2.04) | - | 4 (2.05) | 2 (0.95) | - | - |
| Immunization (vaccination) | 11 (17.74) | 9 (15) | 13 (7.18) | 13 (7.3) | 4 (8.16) | 6 (12.77) | 14 (7.18) | 18 (8.57) | 13 (13.54) | 8 (8.08) |
| **Reference service used^d^** |  |  |  |  |  |  |  |  |  |  |
| Primary Health Care | 51 (82.26) | 53 (88.33) | 106 (58.56) | 119 (66.85) | 31 (63.27) | 24 (51.06) | 87 (44.62) | 77 (36.67) | 64 (66.67) | 63 (63.64) |
| Public Emergency Care Units | 27 (43.55) | 37 (61.67) | 116 (64.09) | 124 (69.66) | 40 (81.63) | 44 (93.62) | 126 (64.62) | 141 (67.14) | 42 (43.75) | 55 (55.56) |
| Emergency/emergency care in public hospitals | 30 (48.39) | 36 (60) | 62 (34.25) | 47 (26.4) | 22 (44.9) | 17 (36.17) | 71 (36.41) | 81 (38.57) | 48 (50) | 47 (47.47) |
| Private services | 11 (17.74) | 18 (30) | 18 (9.94) | 38 (21.34) | 9 (18.36) | 11 (23.41) | 16 (8.22) | 34 (16.19) | 21 (21.87) | 41 (41.41) |
| **Perform gynecological and family planning consultations^d^** | 21 (33.87)* | 34 (57.63) | 77 (42.54)* | 103 (57.87) | 25 (51.02) | 17 (36.17) | 67 (34.36)** | 141 (67.14) | 47 (48.96)* | 68 (68.69) |
| **Last gynecological appointment <2 weeks ago** | 3 (14.29) | 5 (15.15) | 11 (14.67)* | 4 (3.92) | 6 (24) | 1 (5.88) | 10 (14.93)* | 10 (7.09) | 9 (19.15)* | 1 (1.49) |
| **Obstetric history** | | | | | | | | | | |
| **Previous pregnancies** | 11 (17.74)* | 21 (35) | 32 (17.68)** | 62 (34.83) | 6 (12.24) | 2 (4.26) | 25 (12.82)** | 70 (33.33) | 18 (18.75) | 28 (28.28) |
| **Repetition**  **gestational** | 16 (25.80)* | 21 (35) | 41 (22.7)* | 68 (38.20) | 6 (12.24) | 5 (10.63) | 34 (17.44)** | 74 (35.24) | 20 (20.83) | 29 (29.29) |
| **Number of previous pregnancies** | 1 (1 - 1.5) | 1 (1 - 2) | 1 (1 - 1) | 1 (1 - 1) | 1 (1-1.75) | 1 (1-1) | 1 (1 - 1) | 1 (1 - 2) | 1 (1 - 1) | 1 (1 - 2) |
| **Had a miscarriage** | 3 (4.84) | 8 (13.33) | 10 (5.52) | 24 (13.48) | 4 (8.16) | 1 (2.13) | 7 (19.8) | 23 (11) | 6 (6.25) | 11 (11.11) |
| **Age of first child** | 15.82 (1.72)** | 21.57 (2.46) | 15 (15 - 17)** | 22 (21 - 23) | 16 (15.25-17.5) | 21 (20.5-21.5) | 16 (15 - 17)** | 22 (20.25 - 23) | 15 (15 - 16)** | 21 (20 - 22) |
| **They used contraception before pregnancy** | 46 (74.19) | 43 (71.67) | 108 (59.67) | 120 (67.42) | 36 (73.47) | 36 (73.6) | 90 (46.15) | 116 (55.24) | 43 (44.79) | 51 (51.52) |
| **Contraceptive method used** |  |  |  |  |  |  |  |  |  |  |
| Male condom | 13 (20.97) | 17 (28.33) | 17 (9.39) | 24 (13.48) | 16 (32.65) | 12 (25.53) | 29 (14.87) | 43 (20.48) | 24 (25) | 28 (28.28) |
| Female condom | - | - | 2 (1.1) | 3 (1.69) |  |  | - | 2 (0.95) | THAT | 2 (2.02) |
| Pill | 23 (37.1) | 27 (45) | 61 (33.7) | 76 (42.7) | 19 (38.78) | 31 (65.96) | 34 (17.44) | 44 (20.95) | 21 (21.88) | 18 (18.18) |
| Injectable | 24 (38.71) | 14 (23.33) | 45 (24.86) | 35 (19.66) | 11 (22.45) | 3 (6.38) | 41 (21.03) | 61 (29.05) | 14 (14.58) | 24 (24.24) |
| Intrauterine device (IUD) | - | 1 (1.67) | - | 2 (1.12) | - | 2 (4.26) | 1 (0.51) | 1 (0.48) | THAT | 2 (2.02) |
| Subdermal implant | 1 (1.61) | 3 (5) | - | - | - | - | - | - |  |  |
| **Pregnancy history** | | | | | | | | | | |
| ***Number of prenatal consultations*^d^** | | | | | | | | | | |
| <3 queries | - | 1 (1.69) | 0 (0) | 2 (1.16) | 1 (2.04) | 1 (2.27) | 7 (3.65)* | 0 (0) | 6 (6.25)* | 0 (0) |
| 3 to 5 consultations | 4 (6.78) | 5 (8.47) | 16 (8.84) | 14 (8.14) | 15 (30.61) | 5 (11.36) | 33 (17.19)* | 28 (13.53) | 18 (18.75)* | 9 (9.18) |
| 6 consultations | 4 (6.78) | 4 (6.78) | 4 (2.21) | 9 (5.23) | 5 (10.2) | 5 (11.36) | 24 (12.5)* | 24 (11.59) | 16 (16.67)* | 9 (9.18) |
| >6 | 51 (86.44) | 49 (83.05) | 161 (88.95) | 147 (85.47) | 28 (57.14) | 33 (75) | 128 (66.67)* | 155 (74.88) | 56 (58.33)* | 80 (81.63) |
| First prenatal consultation (week of pregnancy)**^d^** | 9 (5 - 16)* | 8 (5 - 10) | 8 (4 - 12)* | 6 (4 - 9.25) | 10 (6 - 16) | 8 (4 - 12) | 8.5 (5 - 15)* | 8 (4 - 12) | 12 (4 - 16)* | 7.5  (5 - 10.25) |
| ***Substance use during pregnancy*** | | | | | | | | | | |
| Alcoholic beverage**^d^** | 14 (22.6) | 9 (15) | 24 (13.26) | 21 (11.78) | 7 (14.29) | 6 (12.77) | 25 (12.82) | 19 (9.05)) | 12 (12.5) | 7 (7.07) |
| Smoke | 4 (6.45) | 6 (9.68) | 11 (6.08) | 13 (7.30) | 4 (8.16) | 2 (4.26) | 10 (5.12) | 5 (2.38) | 3 (3.13) | - |
| Illicit substances | 2 (3.33) | 3 (5) | 1 (0.55) | 4 (2.24) | - | - | 5 (2.56) | 4 (1.90) | 2 (2.08) | 2 (2.02) |
| ***Complications during pregnancy*** | | | | | | | | | | |
| Gestational hypertension**^d^** | 17 (27.87) | 23 (38.33) | 42 (23.20) | 47 (26.40) | 11 (22.92) | 15 (32.61) | 33 (16.92*)* | 64 (30.48) | 20 (20.83) | 27 (27.27) |
| Pre eclampsia**^d^** | 8 (13.56) | 7 (11.67) | 13 (7.3)* | 26 (14.69) | 6 (12.5) | 5 (11.36) | 15 (7.81) | 24 (11.48) | 8 (8.33) | 11 (11.11) |
| Gestational diabetes**^d^** | 12 (19.35) | 16 (26.67) | 13 (7.18) | 25 (14.04) | 4 (8.16) | 5 (10.87) | 9 (4.62)* | 31 (14.76) | 6 (6.25) | 9 (9.09) |
| Anemia**^d^** | 24 (38.71) | 19 (31.67) | 81 (45)* | 61 (34.27) | 12 (24.49) | 11 (23.4) | 109 (55.90)** | 74 (35.24) | 50 (52.08) | 38 (38.78) |
| ***Type of labor*** | | | | | | | | | | |
| Spontaneous | 27 (43.55) | 20 (33.33) | 105 (58.01)** | 67 (37.64) | 20 (40.82) | 13 (27.66) | 113 (57.95)** | 72 (34.29) | 45 (46.88) | 37 (37.37) |
| Induced | 22 (35.48) | 22 (36.67) | 34 (18.78) | 47(26.4) | 6 (12.24) | 3 (6.38) | 13 (6.67) | 22 (10.48) | 4 (4.17) | 7 (7.07) |
| Caesarean section | 13 (20.97) | 18 (30) | 42 (23.2) | 64 (35.96) | 23 (49.94) | 31 (65.96) | 69 (35.38) | 116 (55.24) | 47 (48.96) | 55 (55.56) |
| ***Prematurity*** |  |  |  |  |  |  |  |  |  |  |
| Gestational age**^d^** | 39 (38 - 40) | 39 (38 - 40.75) | 39 (38 - 40) | 39 (38 - 41) | 38.5 (38 - 39) | 39 (37 - 40) | 39 (38 - 40) | 39 (38 - 40) | 39 (36.5 - 40) | 39 (37 - 40) |
| ***Prematurity classification*^d^** |  |  |  |  |  |  |  |  |  |  |
| Very premature | 1 (1.69) | 0 (0) | 2 (1.18) | 2 (1.15) | 2 (4.17) | 1 (2.22) | 3 (1.6) | 4 (1.91) | 3 (3.3) | 4 (4.17) |
| Moderate premature | 7 (11.86) | 4 (6.9) | 27 (15.88) | 16 (9.2) | 7 (14.58) | 8 (17.78) | 17 (9.04) | 24 (11.48) | 20 (21.98) | 17 (17.71) |
| Not premature | 51 (86.44) | 54 (93.1) | 141 (82.94) | 156 (89.66) | 39 (81.25) | 36 (80) | 168 (89.36) | 181 (86.6) | 68 (74.73) | 75 (78.12) |
| **Birth weight^d^** |  |  |  |  |  |  |  |  |  |  |
| Weight (kg) | 3.27 (2.9 - 3.68) | 3.4125 (3.14 - 3.68) | 3 (2.64 - 3.34)** | 3.2575 (3 - 3.7) | 3.3 (2.8 - 3.54) | 3.195 (2.75 - 3.47) | 3.165 (2.77 - 3.5) | 3.1475 (2.8 - 3.5) | 3.1 (2.6 - 3.4) | 3.2 (2.7- 3.52) |
| **Classification^d^** |  |  |  |  |  |  |  |  |  |  |
| Extremely low weight | 1 (1.64) | - | 1 (0.58) | - | - | - | - | - | 2 (2.08) | 1 (1.04) |
| Low weight | 6 (9.84) | 6 (10.34) | 28 (16.37) | 16 (9.2) | 4 (8.51) | 8 (17.39) | 24 (13.26) | 24 (11.65) | 19 (19.79) | 13 (13.54) |
| Not underweight | 54 (88.52) | 52 (89.66) | 142 (83.04) | 158 (90.8) | 43 (91.49) | 38 (82.61) | 157 (86.74) | 182 (88.35) | 75 (78.12) | 82 (85.42) |
| **Legend:** Categorical variables: descriptive in terms of n(%);  Numerical variables: Mean (SD) for normally distributed variables (Shapiro-Wilk test; p>0.05); Median (Q1-Q3) for asymmetric variables (Shapiro-Wilk test; p<0.05)  ^a^ Sample number of teenage mothers in Brazil and by region: Brazil: n=583; South: n=62; Southeast: n= 181; Midwest: n= 49; Northeast: n= 195; North: n= 96.  ^b^Sample number of young adult mothers in Brazil and by region: Brazil: n=594; South: n=60; Southeast: n= 178; Midwest: n= 47; Northeast: n= 210; North: n= 99.  ^c^We only consider the alternatives with the highest number of responses.  **^d^**Variables in which the number of responses differs from the number of participants (Supplementary material Table S5).  * Statistically significant estimate; p<0.05  ** Statistically significant estimate; p<0.001 | | | | | | | | | | |

**Table S3**. Domains Psychological of teenage mothers and young adult mothers in the five regions of Brazil.

| **Variable** | **South region** | | **Southeast region** | | **Midwest region** | | **Northeast Region** | | **North region** | |
| --- | --- | --- | --- | --- | --- | --- | --- | --- | --- | --- |
|  | Teen Moms^a^ | Young adult mothers^b^ | Teen Moms^a^ | Young adult mothers^b^ | Teen Moms^a^ | Young adult mothers^b^ | Teen Moms^a^ | Young adult mothers^b^ | Teen Moms^a^ | Young adult mothers^b^ |
| **Pregnancy factors** | | | | | | | | | | |
| ***Reason why she became a mother:*** |  |  |  |  |  |  |  |  |  |  |
| Without wanting to**^d^** | 37 (59.68) | 29 (48.33) | 114 (63.33)* | 89 (50) | 31 (63.27) | 21 (44.68) | 133 (68.21)* | 118 (56.19) | 61 (63.54) | 49 (50) |
| Because I wanted to be a mother**^d^** | 29 (46.77) | 38 (63.33) | 80 (44.2)* | 110 (61.8) | 16 (32.65) | 25 (53.19) | 61 (31.28)** | 111 (53.11) | 45 (46.88) | 61 (61.62) |
| Because I wanted to have another child | 5 (8.06) | 13 (21.67) | 14 (7.73)* | 36 (20.22) | 3 (6.12) | 0 (0) | 16 (8.21)* | 50 (23.81) | 11 (11.46)* | 23 (23.23) |
| Because I wanted to build a family**^d^** | 25 (40.32)* | 38 (63.33) | 83 (45.86)** | 114 (64.77) | 19 (38.78) | 27 (57.45) | 74 (37.95)** | 121 (57.62) | 47 (48.96)* | 65 (65.66) |
| Because I wanted to leave my parents'/guardians' home | 6 (9.68) | 4 (40) | 25 (13.81) | 15 (8.43) | 1 (2.04) | 2 (4.26) | 21 (10.77) | 16 (7.62) | 22 (22.92)** | 4 (4.04) |
| Because I wanted to get married | 13 (20.97) | 9 (15) | 26 (14.36) | 38 (21.35) | 5 (10.2) | 6 (12.77) | 25 (12.82) | 36 (17.14) | 22 (22.92) | 19 (19.19) |
| Because I thought I would be more respected after becoming a mother | 6 (9.68) | 9 (15) | 26 (14.36) | 17 (9.55) | 5 (10.2) | 1 (2.13) | 35 (17.95)* | 17 (8.1) | 21 (21.88)* | 9 (9.09) |
| Because I didn't know how to avoid having children | 8 (12.9)* | 1 (1.67) | 21 (11.6)* | 6 (3.37) | 4 (8.16) | 0 (0) | 55 (28.21)** | 15 (7.14) | 31 (32.29)** | 3 (3.03) |
| Because I couldn't afford contraceptives | 5 (8.06) | 0 (0) | 8 (4.42) | 3 (1.69) | 1 (2.04) | 0 (0) | 30 (15.38)* | 12 (5.71) | 15 (15.62)* | 2 (2.02) |
| Because married early | 20 (32.26)* | 5 (8.33) | 34 (18.78)* | 14 (7.87) | 14 (28.57)* | 2 (4.26) | 40 (20.51)* | 23 (10.95) | 23 (23.96)* | 8 (8.08) |
| Because there was no other option**^d^** | 2 (3.23) | 0 (0) | 9 (4.97) | 8 (4.49) | 2 (4.17) | 0 (0) | 19 (9.74)* | 7 (3.33) | 14 (14.58)* | 2 (2.02) |
| Because it was a life project**^d^** | 16 (25.81) | 25 (41.67) | 39 (21.55)** | 92 (51.69) | 12 (24.49) | 20 (42.55) | 56 (28.87)** | 98 (46.67) | 22 (22.92)** | 50 (50.51) |
| Because the husband/partner wanted to have children soon**^d^** | 11 (17.74) | 9 (15) | 29 (16.02) | 36 (20.34) | 8 (16.33) | 11 (23.4) | 33 (16.92)* | 55 (26.19) | 18 (18.75) | 21 (21.21) |
| Because the partner didn't want to use a condom | 16 (25.81) | 9 (15) | 39 (21.55) | 25 (14.04) | 11 (22.45) | 4 (8.51) | 51 (26.15) | 43 (20.48) | 26 (27.08) | 20 (20.2) |
| Because I didn't know where to get contraceptives**^d^** | 5 (8.06) | 0 (0) | 6 (3.31) | 5 (2.81) | 3 (6.12) | 0 (0) | 21 (10.77)** | 4 (1.9) | 13 (13.54)* | 2 (2.04) |
| Because the contraceptive failed**^d^** | 17 (27.42) | 12 (20) | 57 (31.49) | 40 (22.47) | 19 (38.78) | 11 (23.4) | 55 (28.35) | 47 (22.38) | 24 (25) | 20 (20.2) |
| ***Feel supported by the child's father*** |  |  |  |  |  |  |  |  |  |  |
| Affectively/emotionally**^d^** | 46 (74.19) | 45 (75) | 126 (69.61)* | 150 (84.27) | 35 (71.43) | 40 (86.96) | 147 (75.77) | 174 (82.86) | 71 (75.53) | 81 (81.82) |
| Routine | 37 (59.68) | 43 (71.67) | 120 (66.3) | 130 (73.03) | 33 (67.35) | 32 (68.09) | 128 (65.64) | 156 (74.29) | 65 (67.71) | 75 (75.76) |
| Financially**^d^** | 48 (77.42) | 48 (80) | 129 (71.67)** | 156 (87.64) | 40 (81.63) | 39 (82.98) | 157 (80.51) | 179 (85.24) | 76 (79.17)* | 91 (91.92) |
| ***Changes that occurred in life after pregnancy:*** |  |  |  |  |  |  |  |  |  |  |
| Your life has become more difficult | 44 (70.97)* | 26 (43.33) | 120 (66.3) | 105 (58.99) | 31 (63.27) | 29 (61.7) | 130 (66.67) | 127 (60.48) | 57 (59.38) | 58 (58.59) |
| Your life has become more organized | 31 (50) | 34 (56.67) | 101 (55.8) | 82 (46.07) | 27 (55.1) | 19 (40.43) | 119 (61.03) | 118 (56.19) | 70 (72.92)* | 57 (57.58) |
| Became more respected**^d^** | 38 (61.29)* | 24 (40) | 112 (62.22)** | 71 (40.57) | 23 (46.94) | 16 (34.04) | 122 (62.56) | 125 (59.52) | 59 (61.46) | 61 (61.62) |
| Your relationship with your husband/partner has improved**^d^** | 28 (45.16) | 35 (59.32) | 88 (49.16)* | 109 (61.24) | 22 (44.9) | 26 (55.32) | 116 (59.49)* | 146 (69.52) | 58 (61.05) | 67 (67.68) |
| Your husband/partner abandoned you**^d^** | 16 (25.81) | 13 (21.67) | 48 (26.67)* | 26 (14.69) | 14 (28.57) | 5 (10.64) | 39 (20) | 28 (13.33) | 28 (29.17) | 19 (19.19) |
| She was rejected by her family**^d^** | 5 (8.06) | 1 (1.67) | 13 (7.18) | 9 (5.06) | 3 (6.12) | 1 (2.13) | 15 (7.69) | 10 (4.76) | 15 (15.79)* | 4 (4.04) |
| Dropped out of school/technical course/college | 34 (54.84)* | 9 (15) | 72 (39.78)** | 23 (12.92) | 21 (42.86)** | 5 (10.64) | 91 (46.67)** | 21 (10) | 39 (40.62)** | 17 (17.17) |
| She wanted to study to give her baby a good future | 55 (88.71) | 51 (85) | 174 (96.13)** | 136 (76.4) | 46 (93.88) | 38 (80.85) | 187 (95.9)** | 176 (83.81) | 94 (97.92)* | 88 (88.89) |
| He now had a reason to live**^d^** | 11 (17.74) | 3 (5) | 25 (13.81) | 25 (14.2) | 7 (14.29) | 6 (12.77) | 40 (20.51)* | 21 (10) | 21 (21.88) | 15 (15.15) |
| It was the worst period of his life**^d^** | 48 (77.42) | 49 (81.67) | 152 (83.98) | 143 (80.79) | 39 (81.25) | 37 (78.72) | 162 (83.51) | 176 (83.81) | 79 (82.29) | 81 (81.82) |
| Difficulty getting and/or staying at work | 37 (59.68) | 28 (46.67) | 101 (55.8)* | 70 (39.33) | 28 (57.14) | 20 (42.55) | 114 (58.46)** | 75 (35.72) | 63 (65.62)* | 41 (41.41) |
| Created new friendships or got closer to women who are also mothers**^d^** | 22 (35.48) | 25 (41.67) | 78 (43.33)* | 101 (56.74) | 13 (26.53) | 22 (46.81) | 82 (42.05) | 107 (50.95) | 40 (41.67) | 52 (52.53) |
| **Mental health** | | | | | | | | | | |
| ***Has a doctor or mental health professional (such as a psychiatrist or psychologist) ever told you that you had depression or anxiety?*** | | | | | | | | | | |
| Yes, before pregnancy in 2021/2022 | 16 (25.81) | 14 (23.33) | 22 (12.15) | 28 (15.73) | 12 (24.49) | 12 (25.53) | 23 (11.79) | 25 (11.9) | 15 (15.62) | 14 (14.14) |
| Yes, during the 2021/2022 pregnancy | 6 (9.68) | 4 (6.67) | 18 (9.94) | 15 (8.43) | 4 (8.16) | 2 (4.26) | 11 (5.64) | 12 (5.71) | 5 (5.21) | 9 (9.09) |
| ***Use of medication for anxiety during pregnancy*** | 2 (3.23) | 3 (5) | 2 (1.10) | 5 (2.81) | 3 (6.12) | 0 (0) | 4 (2.05) | 5 (2.38) | 2 (2.08) | 2 (2.02) |
| ***Use of medication for depression during pregnancy*** | 0 (0) | 3 (5) | 1 (0.55) | 2 (1.12) | 0 (0) | 0 (0) | 1 (0.51) | 3 (1.43) | 1 (1.04) | 0 (0) |
| ***Prevalence of depression and anxiety levels at the time of the interview*** | | | | | | | | | | |
| Depression | 38 (61.29) | 29 (48.33) | 112 (61.88) | 90 (50.56) | 34 (9.42) | 30 (10) | 115 (31.86) | 102 (34) | 62 (17.17) | 49 (16.33) |
| Anxiety | 41 (66.13) | 30 (50) | 118 (65.19) | 87 (48.87) | 32 (8.6) | 27 (8.79) | 119 (31.99) | 109 (35.5) | 62 (16.67) | 50 (16.29) |
| **Legend:** Categorical variables: descriptive in terms of n(%);  Numerical variables: Mean (SD) for normally distributed variables (Shapiro-Wilk test; p>0.05); Median (Q1-Q3) for asymmetric variables (Shapiro-Wilk test; p<0.05)  ^a^ Sample number of teenage mothers in Brazil and by region: Brazil: n=583; South: n=62; Southeast: n= 181; Midwest: n= 49; Northeast: n= 195; North: n= 96.  ^b^Sample number of young adult mothers in Brazil and by region: Brazil: n=594; South: n=60; Southeast: n= 178; Midwest: n= 47; Northeast: n= 210; North: n= 99.  ^c^Multiple choice variables, where we only consider the alternatives with the greatest number of answers.  ^d^Variables in which the number of responses differs from the number of participants (Supplementary material Table S5).  * Statistically significant estimate; p<0.05  ** Statistically significant estimate; p<0.001 | | | | | | | | | | |

**Table S4**. Domainssocial of teenage mothers and young adult mothers in the five regions of Brazil.

| **Variable** | **South region** | | **Southeast region** | | **Midwest region** | | **Northeast Region** | | **North region** | |
| --- | --- | --- | --- | --- | --- | --- | --- | --- | --- | --- |
|  | Teen Moms^a^ | Young adult mothers^b^ | Teen Moms^a^ | Young adult mothers^b^ | Teen Moms^a^ | Young adult mothers^b^ | Teen Moms^a^ | Young adult mothers^b^ | Teen Moms^a^ | Young adult mothers^b^ |
| **Sociodemographic data** | | | | | | | | | | |
| ***Participate in government social programs*^d^** | 41 (66.13) | 32 (53.33) | 132 (73.33)** | 97 (54.59) | 32 (66.67)* | 19 (40.43) | 140 (71.79) | 138 (65.71) | 58 (61.05) | 68 (68.69) |
| ***Family income (R$)*^d^** | 1290 (1200 - 1800) | 1500 (1206 - 2250) | 1300 (800 - 1925)* | 1400 (1200 - 2275) | 1552 (1302 - 2425) | 1800 (1302 - 3000) | 1212 (650 - 1800)* | 1400 (1000 - 2000) | 1212 (1000 - 2000)* | 1800 (1200 - 2500) |
| ***Age of the child's father:*** | 21 (19 - 24)** | 27 (24 - 30.5) | 23 (21 - 25)** | 29 (26 - 33) | 22 (20 - 24)** | 28 (25 - 32) | 22 (19 - 25)** | 29 (25 - 33) | 22 (20 - 25)** | 29 (25 - 33) |
| ***Currently studying*** | 15 (24.19)* | 3 (5) | 36 (19.89)* | 14 (7.87) | 12 (24.49) | 4 (8.51) | 36 (18.46)* | 16 (7.62) | 21 (21.88) | 12 (12.12) |
| ***Reasons for not studying*^d^** | | | | | | | | | | |
| Pregnancy/Being a mother | 30 (48.39) | 30 (50) | 58 (32.04) | 38 (21.35) | 21 (42.86) | 13 (27.66) | 105 (53.85) | 67 (31.9) | 47 (48.96) | 44 (44.44) |
| Finished high school | 3 (4.84) | 9 (15) | 26 (14.36) | 71 (39.89) | 3 (6.12) | 7 (14.89) | 35 (17.95) | 60 (28.57) | 13 (13.54) | 24 (24.24) |
| Lack of time | 12 (19.35) | 15 (25) | 27 (14.92) | 30 (16.85) | 8 (16.33) | 5 (10.64) | 11 (5.64) | 37 (17.62) | 21 (21.88) | 20 (20.2) |
| **Main type of institution where the interviewees studied^d^** | | | | | | | | | | |
| Public school | 57 (93.44) | 50 (83.33) | 174 (96.13)* | 145 (81.46) | 47 (95.92) | 31 (65.96) | 187 (95.9)* | 149 (70.95) | 89 (93.68)* | 54 (56.84) |
| Private School | 2 (3.28) | 2 (3.33) | 3 (1.66) | 11 (6.18) | 1 (2.04) | 1 (2.13) | 4 (2.05) | 28 (13.33) | 3 (3.16) | 8 (8.42) |
| **Stopped studying during pregnancy** | 36 (58.06)* | 17 (28.33) | 90 (49.72)** | 34 (19.1) | 23 (46.94)* | 7 (14.89) | 103 (52.82)* | 27 (12.86) | 48 (50)* | 23 (23.23) |
| ***How long did you stop studying during pregnancy?*** | | | | | | | | | | |
| Up to 6 months | 5 (13.89) | 3 (17.65) | 20 (22.73) | 3 (8.82) | 7 (30.43) | 0 | 34 (33.33) | 9 (33.33) | 17 (36.17) | 4 (17.39) |
| More than 6 months | 31 (86.11) | 14 (82.35) | 68 (77.27) | 31 (91.18) | 16 (69.57) | 7 (100) | 68 (66.67) | 18 (66.67) | 30 (63.83) | 19 (82.61) |
| ***Employment status*^d^** | | | | | | | | | | |
| Working | 7 (11.29)** | 18 (30) | 37 (20.44)** | 54 (30.51) | 14 (28.57)** | 15 (31.91) | 23 (11.79)* | 60 (28.57) | 12 (12.5)* | 23 (23.23) |
| Unemployed | 25 (40.32) | 37 (61.67) | 97 (53.59) | 114 (64.41) | 19 (38.78) | 31 (65.96) | 98 (50.26) | 130 (61.9) | 45 (46.88) | 58 (58.59) |
| Never worked | 30 (48.39) | 5 (8.33) | 47 (25.97) | 9 (5.08) | 16 (32.65) | 1 (2.13) | 74 (37.95) | 20 (9.52) | 39 (40.62) | 18 (18.18) |
| ***I was working when my son was born*** | 6 (18.75)* | 23 (41.82) | 23 (17.16)** | 79 (46.75) | 15 (45.45) | 25 (54.35) | 23 (19.01)* | 70 (36.84) | 13 (22.81)* | 35 (43.21) |
| ***Taken leave from work*** | 4 (6.45) | 21 (35.00) | 13 (56.53) | 58 (73.42) | 7 (46.67) | 25 (100) | 8 (69.58) | 43 (61,42) | 9 (69,22) | 19 (53,92) |
| ***Transgenerational Factors*** | | | | | | | | | | |
| ***Age at which the maternal mother had her first biological child*^d^** | 17 (15 - 19.75) | 18 (16 - 21.25) | 17 (16 - 20)* | 19 (17 - 22) | 17 (15 - 19) | 18 (16 - 21) | 17 (15.25 - 19)* | 19 (16 - 21) | 17 (15.25 - 19) | 18 (16 - 21) |
| ***How many children did the mother have?*^d^** | 4 (3 - 6) | 4 (3 - 5) | 4 (3 - 5) | 3 (2 - 5) | 4 (3 - 6)* | 3 (2.5 - 4) | 3 (2 - 5) | 3 (2 - 4) | 4 (3 - 6) | 4 (3 - 5) |
| ***Maternal grandmother had children during adolescence*^d^** | 26 (56.52) | 27 (65.85) | 91 (68.42) | 93 (72.66) | 32 (78.05) | 26 (76.47) | 105 (76.64) | 133 (79.648) | 58 (69.05) | 59 (77.63) |
| ***Paternal grandmother had children during adolescence*^d^** | 20 (58.82) | 23 (69.70) | 70 (66.04) | 79 (70.54) | 22 (61.11)* | 26 (86.67) | 81 (73.64) | 105 (73.94) | 51 (69.86) | 48 (77.42) |
| **Legend:** Categorical variables: descriptive in terms of n(%);  Numerical variables: Mean (SD) for normally distributed variables (Shapiro-Wilk test; p>0.05); Median (Q1-Q3) for asymmetric variables (Shapiro-Wilk test; p<0.05)  ^a^ Sample number of teenage mothers in Brazil and by region: Brazil: n=583; South: n=62; Southeast: n= 181; Midwest: n= 49; Northeast: n= 195; North: n= 96.  ^b^Sample number of young adult mothers in Brazil and by region: Brazil: n=594; South: n=60; Southeast: n= 178; Midwest: n= 47; Northeast: n= 210; North: n= 99.  ^c^We only consider the alternatives with the highest number of responses.  ^d^Variables in which the number of responses differs from the number of participants (Supplementary material Table S5).  * Statistically significant estimate; p<0.05  ** Statistically significant estimate; p<0.001 | | | | | | | | | | |

| **Table S5.** Number per variable, in which we did not obtain 100% of the responses. | | | |
| --- | --- | --- | --- |
| Variable | N total response | N in the group teenagers mothers | N in the young adult mothers group |
| ***Biological domain*** | | | |
| ***Health comorbidities*** | 1341 | 655 | 686 |
| ***Medication use*** | 1176 | 583 | 593 |
| ***Visited a health service <2 weeks ago*** | 1165 | 583 | 594 |
| ***Main reasons for consulting health services^c^*** | 819 | 644 | 648 |
| ***Reference service used*** | 1175 | 1332 | 1427 |
| ***Perform gynecological and family planning consultations*** | 1177 | 583 | 594 |
| ***Number of prenatal consultations*** | 1157 | 424 | 580 |
| ***First prenatal consultation (week of pregnancy)*** | 1095 | 531 | 564 |
| ***Use of alcoholic beverages during pregnancy*** | 1049 | 523 | 526 |
| ***Complications during pregnancy*** |  |  |  |
| Gestational hypertension | 1174 | 581 | 593 |
| Pre eclampsia | 1162 | 573 | 589 |
| Gestational diabetes | 1176 | 583 | 589 |
| Anemia | 1174 | 581 | 593 |
| Gestational age (week) | 1138 | 556 | 582 |
| ***Prematurity classification*** | 1138 | 556 | 582 |
| ***Birth weight*** | 1136 | 556 | 580 |
| ***Birth weight classification*** | 1136 | 556 | 580 |
| ***Psychological domain*** | | | |
| ***Reasons why she became a mother*** |  |  |  |
| Without wanting to | 1175 | 582 | 593 |
| Because I wanted to be a mother | 1176 | 583 | 593 |
| Because I wanted to build a family | 1175 | 583 | 592 |
| Because there was no other option | 1176 | 582 | 594 |
| Because it was a life project | 1176 | 582 | 594 |
| Because the husband/partner wanted to have children soon | 1176 | 583 | 593 |
| Because I didn't know where to get contraceptives | 1176 | 583 | 593 |
| Why the contraceptive failed | 1176 | 582 | 594 |
| ***Support from the child's father: affectively/emotionally*** | 1173 | 580 | 593 |
| ***Support from the child's father: financial*** | 1176 | 582 | 594 |
| ***Changes that occurred in life after pregnancy*** |  |  |  |
| Became more respected | 1173 | 582 | 591 |
| Your relationship with your husband/partner has improved | 1173 | 580 | 593 |
| Your husband/partner abandoned you | 1175 | 582 | 593 |
| She was rejected by her family | 1176 | 582 | 594 |
| He now had a reason to live | 1175 | 583 | 592 |
| It was the worst period of his life | 1174 | 581 | 593 |
| Created new friendships or got closer to women who are also mothers | 1176 | 582 | 594 |
| ***Social Domain*** | | | |
| ***Participate in government social programs*** | 1174 | 580 | 594 |
| ***Family income (R$)*** | 1018 | 471 | 547 |
| ***Reasons for not studying*** | 1007 | 562 | 701 |
| ***Main type of institution where the interviewees studied*** | 1171 | 581 | 590 |
| ***Employment status*** | 1176 | 583 | 593 |
| ***Age at which the mother had her first biological child*** | 973 | 491 | 482 |
| ***How many children did the mother have*** | 1176 | 583 | 593 |
| ***Maternal grandmother had children as teenagers*** | 887 | 441 | 446 |
| ***Paternal grandmother had children when she was a teenager*** | 738 | 359 | 379 |
